# Tissue Engineering Using Vascular Organoids From Human Pluripotent Stem Cell Derived Mural Cell Phenotypes

**DOI:** 10.3389/fbioe.2020.00278

**Published:** 2020-04-17

**Authors:** Maria Markou, Dimitrios Kouroupis, Fotios Badounas, Athanasios Katsouras, Athena Kyrkou, Theodore Fotsis, Carol Murphy, Eleni Bagli

**Affiliations:** ^1^Laboratory of Biological Chemistry, Medical School, University of Ioannina, Ioannina, Greece; ^2^Foundation for Research and Technology-Hellas, Department of Biomedical Research, Institute of Molecular Biology and Biotechnology, Ioannina, Greece; ^3^Transgenic Technology Laboratory, Inflammation Group, Department of Immunology, Hellenic Pasteur Institute, Athens, Greece

**Keywords:** mural cells, tissue engineering, induced pluripotent stem cells, smooth muscle cells, vascularization, vascular organoids, spheroids, regenerative medicine

## Abstract

Diffusion is a limiting factor in regenerating large tissues (100–200 μm) due to reduced nutrient supply and waste removal leading to low viability of the regenerating cells as neovascularization of the implant by the host is a slow process. Thus, generating prevascularized tissue engineered constructs, in which endothelial (ECs) and mural (MCs) cells, such as smooth muscle cells (SMCs), and pericytes (PCs), are preassembled into functional *in vitro* vessels capable of rapidly connecting to the host vasculature could overcome this obstacle. Toward this purpose, using feeder-free and low serum conditions, we developed a simple, efficient and rapid *in vitro* approach to induce the differentiation of human pluripotent stem cells-hPSCs (human embryonic stem cells and human induced pluripotent stem cells) to defined SMC populations (contractile and synthetic hPSC-SMCs) by extensively characterizing the cellular phenotype (expression of CD44, CD73, CD105, NG2, PDGFRβ, and contractile proteins) and function of hPSC-SMCs. The latter were phenotypically and functionally stable for at least 8 passages, and could stabilize vessel formation and inhibit vessel network regression, when co-cultured with ECs *in vitro*. Subsequently, using a methylcellulose-based hydrogel system, we generated spheroids consisting of EC/hPSC-SMC (vascular organoids), which were extensively phenotypically characterized. Moreover, the vascular organoids served as focal starting points for the sprouting of capillary-like structures *in vitro*, whereas their delivery *in vivo* led to rapid generation of a complex functional vascular network. Finally, we investigated the vascularization potential of these vascular organoids, when embedded in hydrogels composed of defined extracellular components (collagen/fibrinogen/fibronectin) that can be used as scaffolds in tissue engineering applications. In summary, we developed a robust method for the generation of defined SMC phenotypes from hPSCs. Fabrication of vascularized tissue constructs using hPSC-SMC/EC vascular organoids embedded in chemically defined matrices is a significant step forward in tissue engineering and regenerative medicine.

**Graphical Abstract:**
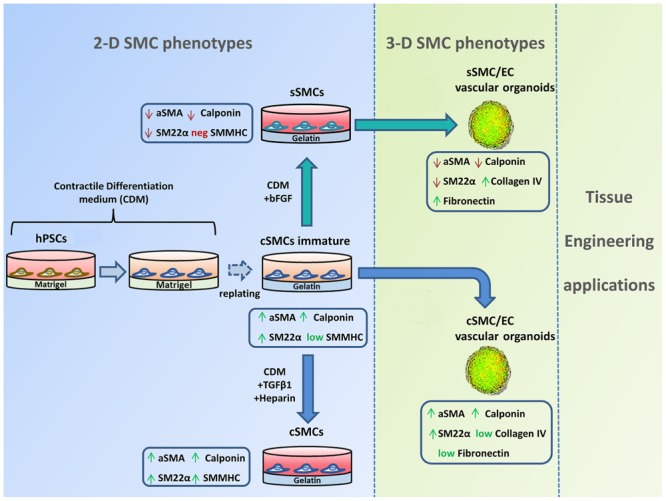
A quick and robust method has been developed to generate both contractile smooth muscle cells (cSMCs) and synthetic SMCs (sSMCs) from human pluripotent stem cells (hPSCs). According to our differentiation protocol, a combination of low serum conditions and subsequent seeding of the cells on gelatin, as ECM coating, can rapidly generate from hPSCs an immature cSMC phenotype (αSMA^high^, Calponinhigh, SM22^high^, SMMHC^+^) that remain stable and without signs of senescence for at least 8 passages. Short treatment with TGFβ1 and heparin induce maturation of the cSMCs seen by the upregulated expression of SMMHC. In contrast, short-term FGF2 exposure of immature cSMCs result in downregulation of αSMA, Calponin, and SM22α expression, consistent with the development of sSMCs. The assembly of cSMC and sSMC subtypes with endothelial cells (ECs) into 3D spheroid co-cultures (vascular organoids), preserve their phenotypic features and serve as focal points for the sprouting of capillary-like structures *in vitro*. Finally, 3D SMC vascular organoid delivery *in vivo* result into rapid generation of a complex and functional vascular network.

## Introduction

Regenerative Medicine is an interdisciplinary field of research and clinical applications, focused on repair, replacement, or regeneration of cells, tissues, or organs to restore impaired function resulting from congenital defects, disease and trauma (Heidary Rouchi and Mahdavi-Mazdeh, 2015). A major requirement for viability and function of the implantable construct is the availability of blood vessels to support its *in vivo* growth. Vascularization remains a critical obstacle in engineering thicker, metabolically demanding organs, such as heart muscle, brain and liver as regenerating tissue over 100–200 μm exceeds the capacity of nutrient supply and waste removal by diffusion, and requires a vascular network (Carmeliet and Jain, 2000; Jain, 2005). It takes several weeks for a scaffold to become fully vascularized *in vivo* (Nillesen et al., 2007), and without a rapid and high level of vascularization of the transplanted grafts, the majority of cells fail to survive the early post-transplantation phase. Therefore, the development of strategies that enhance the angiogenic process represents one of the major research topics in the field of tissue engineering.

The current classical approach is based on the isolation, expansion and seeding of endothelial cells (ECs) onto a suitable scaffold before *in vivo* implantation (Schechner et al., 2000). Although ECs implanted *in vivo* can form an immature vascular network, the ineffective integration of this network into the host vasculature causes regression of the vessels within a few days (Jain et al., 2005; Au et al., 2009). Moreover, the generated capillaries are leaky and unable to properly control permeability, contributing to tissue edema (Hashizume et al., 2000; Melero-Martin et al., 2008). Therefore, a particular challenge for the tissue-engineering community is to induce vascularization of ischemic tissues with blood vessels that are functionally normal. To promote the maturation and stability of nascent vasculatures, ECs must functionally interact with mural cells (MCs), such as vascular smooth muscle cells (vSMCs).

MCs are primarily responsible for stabilization, inhibition of regression, contraction of the vessel as well as production and deposition of extracellular matrix (ECM) proteins (Shepro and Morel, 1993; Chistiakov et al., 2015). Interactions between MCs and ECs are critical in the process of vascular development (Armulik et al., 2005; Regan and Majesky, 2009; Trkov et al., 2010). MCs are composed of vSMC, surrounding larger vessels, such as arteries and veins, and pericytes (PCs), typically surrounding smaller microvessels and capillaries. However, heterogeneities exist within the subtypes (Hedin and Thyberg, 1987; Kusuma and Gerecht, 2013) and the existence of transitional cell phenotypes has recently been suggested in the literature (Holm et al., 2018). As a result, MCs exhibit overlapping marker expression and cannot be distinguished by one marker alone; instead, a combination of markers is required for their identification. Regarding vSMCs, two distinct phenotypes have been identified: synthetic and contractile (Hedin and Thyberg, 1987; Beamish et al., 2010). Both participate in neovascularization, but synthetic vSMCs predominate in the embryo and in diseased or injured adult vessels, while contractile vSMCs predominate in healthy adult vessels. In this context, understanding distinctions between MCs and the molecular mechanisms underlining their phenotypic stability and plasticity, will enable improved therapeutics in a tissue-specific manner. However, although the role of MCs in engineering vascularized constructs for therapeutic applications is unquestionable (Wanjare et al., 2013b; Dar and Itskovitz-Eldor, 2015), their dynamic phenotypic nature has not been extensively studied mainly due to limitations of isolation/expansion and phenotypic plasticity during *in vitro* culture of primary MCs (Cathery et al., 2018).

Selection of the suitable cell source for vascular tissue engineering as well as development of functional capillaries in the fabricated construct are crucial and can be a challenge. Human pluripotent stem cells (hPSCs), including induced PSCs (iPSCs), and embryonic stem cells (ESCs), can differentiate into the three germ layers. They have an unlimited ability to self-renew, making them easy to expand and, despite limitations of the current differentiation procedures (Liu and Zheng, 2019), represent an unlimited source of cells for therapeutic use. The generation of iPSCs, although laborious and expensive, overcomes the ethical problems associated with the clinical use of stem cells and provides the possibility of using autologous cells (Takahashi and Yamanaka, 2006). Using PSCs, the generation of defined phenotypes of MCs can be achieved and offers great potential for studying their plasticity and interactions with ECs. Furthermore, adequate number of cells for tissue engineering applications can be produced, a known obstacle of using primary cells. However, due to the dynamic nature of MCs, one must ensure stability of their phenotype and functionality upon *in vivo* administration.

Over the last few years it has become apparent that when cells are cultured in three-dimension (3D) they adhere to each other via ECM and form natural cell-cell contacts, which transmit physiological information regulating cell growth, migration, differentiation, and survival (Derda et al., 2009; Bhang et al., 2011) resembling the native environment. Spheroidal aggregation has been shown to stabilize ECs and render them responsive to the activities of survival factors (Korff and Augustin, 1998) and, moreover, when spheroids of cultured ECs were embedded in a variety of biomaterials, they served as focal starting points of outgrowing capillary sprouts (Korff et al., 2004; Alajati et al., 2008). In this context, mixed spheroids consisting of ECs and defined populations of MCs, could guarantee the physiological microenvironment for their function and offer the potential for multiple tissue engineering applications.

In the present study, we first induced the differentiation of hESCs and hiPSCs, generated by our group (Kyrkou et al., 2016), to defined SMC subtypes, which were extensively phenotypically and functionally analyzed. The SMCs were then used in a (3D) spheroidal co-culture model with primary human ECs, generating vascular organoids, in order to elucidate the potential of early and robust generation of mature neovessels *in vivo* for tissue engineering applications.

## Materials and Methods

### Cell Culture

#### hPSCs

hiPSCs were generated from human fibroblasts as previously described (Kyrkou et al., 2016), and the H1 hESC line was purchased from Wicell Research Institute (Madison, WI, United States). hPSCs were cultured on six-well tissue culture plates coated with hESC-qualified Matrigel (Corning, 354277) in mTeSR1 medium (StemCell Technologies, 05850) at 37°C and 5% CO_2_. Every 4–6 days, cells were passaged enzymatically using 1 mg/ml dispase (Invitrogen, 17105-041) for 2 min at 37°C. hPSC colonies were then harvested, dissociated into small clumps and replated onto Matrigel-coated 6-well plates (ratio 1:6).

#### Human ECs

ECs from umbilical vein (HUVEC) were cultured in M199 (Gibco) medium supplemented with 20% fetal calf serum (FCS), 47 μg/ml endothelial cell growth supplement (ECGS), 4.7 μ/ml heparin (Sigma) and 1% penicillin-streptomycin as previously described (Bellou et al., 2012).

### Differentiation Protocol

To generate contractile SMCs (cSMCs), hPSCs plated on matrigel were cultured in contractile differentiation medium (CDM) consisting of basal medium (Lonza, PT-3273) supplemented with 2.5% FCS (Gibco, 10270-106) and glutamax (Gibco, 35050) for 9 days with daily medium changes. On day 9, cells were detached enzymatically using 0.05% Trypsin-EDTA (Gibco, 25300-054) and replated on gelatin (0.1% Gelatin-Millipore, ES-006-B) coated dishes and cultured in CDM until confluency (2–3 days). Medium was changed every second day. For generation of a mature phenotype of cSMCs, the differentiated hPSC-cSMCs were induced with 5 ng/ml TGFβ1 (Peprotech) and 50 μ/ml heparin (Sigma, H-3149) in CDM, for 2 days.

To generate synthetic SMCs (sSMCs), hPSC-cSMCs were seeded on gelatin coated dishes for 24 h and subsequently the medium was changed to either CDM supplemented with 2 ng/ml FGF2 (Immunotools, 11343623) or synthetic differentiation medium (SDM) for 48 h. SDM medium consisted of a basal medium (ScienCell, 1201-b) supplemented with 2% FCS, and a combination of growth factors (ScienCell, 1252) 2 ng/ml EGF, 2 ng/ml FGF2 and 2 ng/ml IGF-I.

### Generation of Vascular Organoids/Spheroids

Vascular organoids (spheroids consisted of hPSC-SMC/ECs) and cell spheroids (consisted of ECs) were created using methylcellulose (Sigma, M0512) and the hanging drop technique as previously described (Korff and Augustin, 1998). In brief, each vascular organoid was generated from 1,000 cells at a ratio 1:9 hPSC-SMCs:ECs in 10 μl solution (EGM-2 medium/methylcellulose solution:4:1), cultured in a hanging drop for 2 days at 37°C and 5% CO_2_.

### Immunophenotyping

Flow cytometry analysis of the cells was performed as previously described (Tsolis et al., 2016). Briefly, 2.0 × 10^5^ cells were incubated with FITC/PE/APC-conjugated anti-human primary monoclonal antibodies (Supplementary Table S1). Dead cells were excluded using 2 μg/ml 7-Amino-actinomycin D (7-AAD) viability staining solution (Invitrogen, Thermo Fisher Scientific, Waltham, MA, United States). Background fluorescence was established using isotype controls and data were acquired using CyFlow (Partec, Münster, Germany) collecting a minimum of 20,000 events. The analysis was performed using FlowMax software.

### Western Blot Analysis

Protein extraction was performed from whole cell lysates and quantified with BCA Protein Assay kit (Thermo Scientific, 23225). Samples were prepared, subjected to SDS-PAGE and blotted onto a nitrocellulose membrane, as previously described (Bellou et al., 2012). Specific proteins were detected following incubation with primary antibodies (Supplementary Table S2) and peroxidase-conjugated secondary antibodies (Supplementary Table S2). Quantification of band intensities was performed using Quantity One Analysis software (BIO-RAD).

### Immunofluorescence

Adherent cells: Indirect immunofluorescence on adherent cells was performed as previously described (Bellou, 2012 #68) using primary and secondary antibodies (listed in Materials and Methods Supplementary). Cell nuclei were stained using propidium iodide-PI (Sigma), samples were mounted in moviol-dabco, and images of nine fields were taken on a Leica TCS SP5 confocal microscope using HCX PL APO CS 40 × 1.25 OIL objective.

Vascular organoids/spheroids: Vascular organoids or spheroids consisting of 1,000 cells/spheroid were fixed in 3.7% paraformaldehyde for 1 h at RT, permeabilized with 0.2% Triton-X/0.9% gelatin solution for 1 h, and 0.5% Triton-X/0.9% gelatin solution for 15 min, and incubated with primary antibodies overnight at 4°C (Supplementary Table S2). Next day, the vascular organoids were washed 5x with 0.2% Triton-X and incubated with secondary antibodies for 1 h (Supplementary Table S2). After rinsing 5x with 0.2% Triton-X and incubation with Draq5 (Thermo Fisher Scientific) for 10 min, images were taken on a Leica TCS SP5 confocal microscope using HCX PL APO CS 40 × 1.25 OIL objective. At least 10 vascular organoids or spheroids were analyzed per experiment.

### Quantitative Real Time-PCR (qRT-PCR)

Total RNA was extracted using RNeasy Midi kit (NucleoSpin) according to manufacturer’s instructions, and quantified using NanoDrop^TM^ 1000 Spectrophotometer (Thermo Fisher Scientific). 10ng of RNA were quantified by QuantiTect SYBR Green RT-PCR Kit (Qiagen, 204243) using a LightCycler^®^ 2.0 thermocycler (Roche Diagnostics). For each target transcript primers were selected using DnaStar software (Supplementary Table S3). All samples were analyzed in triplicates per experiment. Expression of the *Smooth Muscle 22-alpha (SM22*α*), CNN1, CD105, Platelet-Derived Growth Factor Receptor Beta (PDGFRB)* and *N G2* transcripts was calculated using a standard curve of RNA isolated from Adipose-derived stem cells. For *Smooth Muscle Myosin Heavy Chain (SMMHC)* mean values were normalized to *Glyceraldehyde-3-Phosphate Dehydrogenase (GAPDH)* and for each transcript the relative expression was calculated using the 2−ΔCT method (Kouroupis et al., 2016).

### Contraction Assay

40,000 hESC-cSMCs attached on 24 well plates were induced to contract with 10^–5^M carbachol (Sigma) in plain DMEM for 30 min at 37°C. Cells were stained with calceinAM (eBioscience, 65-0853). A series of time-lapse images with a 20X (s plan fluor elwd) objective were taken using IncuCyte (IncuCyte ZOOM 2016B). Quantification of the contracted cells/total number of cells was performed in 4 fields/well (6 wells/experiment).

### Cell Proliferation Assay

40,000 hPSC-cSMCs or hPSC-sSMCs were seeded in a 24 well plate in CDM and SDM respectively. After 6 h cells were counted (*t* = 0) or cultured in DMEM 2% FCS. Cell counting was also performed 24 and 48 h post-seeding by the addition of 0.2% trypan blue (Sigma, T815). Average cell number was evaluated from triplicates for each time point and for each cell type.

### Migration Assay (Wound Healing)

The migration of hPSC-cSMCs and hPSC-sSMCs was assessed using a wound healing assay. Cells were cultured to confluence and a “wound gap” was created by scratching a strip on the cell monolayer using a 200 μl pipet tip. Cells were washed with PBS, and cultured in DMEM 0.5% FCS. Optical microscope images of cell migration were taken in a time course every 1 h with a 5X objective. The average number of migrated cells from triplicates was measured with ImageJ software.

### Gelatin Zymography

Zymographic assays were performed to determine matrix metalloproteinase (MMP) activity as previously described (Panopoulou et al., 2005) at cell lysates and conditioned medium (secretome) as well. In brief, hPSC-cSMCs and hPSC-sSMCs were seeded in a 24 well plate in CDM and SDM respectively. After 24 h medium was changed to serum free DMEM medium, and cells were cultured for 72 h. Whole cell lysates were centrifuged at 16,100rcf for 20 min and the concentration of total proteins was quantified with BCA Protein Assay kit. Conditioned media were collected and centrifuged at 1,000rcf for 10 min, to eliminate dead cells. Samples prepared in non-reducing conditions and containing equal amount of total protein were loaded to a gel supplemented with 1 mg/ml gelatin and subjected to SDS-PAGE at 4°C. Regarding the conditioned media, the volume from each sample that was loaded was normalized to protein concentration of the cell lysate. Gel was washed with a solution containing 2.5% Triton X-100, 50 mM Tris-HCL pH7.5, 5 mM CaCL_2_, 1 μM MgCL_2_ and incubated with 1% Triton X-100, 50 mM Tris-HCL pH7.5, 5 mM CaCL_2_, 1 μM MgCL_2_ solution at 37°C overnight. Then whole gel was stained with staining solution (40% methanol, 10% acetic acid, 0.5 g/100 ml Coomassie blue) for 30 min, rinsed with H_2_O, and destained with 40% methanol, 10% acetic acid until bands could clearly be seen. Areas of enzyme activity appeared as white bands against a dark blue background. Gels were scanned, images were modified to be in black and white and then they were inverted so that the areas of enzyme activity appeared black. Quantification of band intensities was performed using Quantity One Analysis software. Alternatively, cell lysate samples prepared as for gelatin zymography were subjected to SDS-PAGE and blotted onto a nitrocellulose membrane. Tubulin was detected (western blot analysis section), in order to confirm equal samples loading.

### Multipotency of hPSC-SMC Subtypes

Cultured hPSC-cSMCs and hPSC-sSMCs were expanded *in vitro* under standard conditions to passage 3 and then subjected to osteogenesis and chondrogenesis differentiation induction protocols, in duplicates, as previously described (Vlaikou et al., 2017).

### *In vitro* Angiogenesis Assay

Monocells: hPSC-cSMCs/sSMCs and ECs were stained with the general membrane staining kit (PKH26, and PKH67 Fluorescent Cell Linker Kits, Sigma-Aldrich) according to manufacturer’s instructions and seeded as monocells at a ratio of 1:9 (hPSC-SMCs:ECs) on a polymerized layer of matrigel in μ-Slide Angiogenesis plates (ibidi, 81501) in triplicates. Images from at least 5 fields/well were taken every 1 h for 48 h with a confocal microscope at 10X magnification. The newly formed network was evaluated using ImageJ software^1^.

Vascular organoids/spheroids: Spheroids and vascular organoids (spheroids consisting of hPSC-SMC/EC) were plated on polymerized matrigel, collagen I (3 mg/ml), collagen I (3 mg/ml) with fibronectin (100 ng/ml) (Benning et al., 2018), and fibrin gel (2 mg/ml) (Nakatsu et al., 2003) in μ-Slide Angiogenesis plates and cultured in EGM-2 medium (1 spheroid/well). Media changes were performed every 2 days and spheroid sprouting was observed daily. Images from at least 3 vascular organoids or spheroids were taken on day 1and 3 using Leica TCS SP5 confocal microscope.

### Tubulogenesis Assay

hPSC-cSMCs and hPSC-sSMCs in duplicates were seeded in the inner surface of ibidi dishes (81156, μ-Dish 35 mm). When confluent, 40,000 HUVECs were added on the cell monolayer and medium was changed to EGM-2 (Lonza, CC-3162). The medium was changed again on day 2. After 4 days cells were fixed and immunofluorescence was performed. Images were taken from the whole plate using Leica TCS SP5 confocal microscope and the formed tube-like network was evaluated with ImageJ software.

### *In vivo* Angiogenesis Plug Assay

Formation of new blood vessels *in vivo* was evaluated using the matrigel plug assay. The experimental protocol was approved by the Committee for Animal Research Studies of the University of Ioannina (No14144). All the experimental procedures were in agreement with European Union directives (EU Directive 2010/63). Appropriate measures were taken to minimize pain or discomfort to the animals. Six to eight weeks old female NSG (NOD/SCID) mice were used. Animals were housed in individually ventilated cages in the animal house facility of University of Ioannina, under a 12 h light/dark cycle, pathogen-free conditions and they had free access to food and water.

ECs spheroids and hPSC-SMC/ECs as vascular organoids (300 spheroids/animal) or as monocells (300,000 cells/animal) were mixed with 200 μl cold matrigel (Corning, 354234), 200 ng/ml vascular endothelial growth factor (VEGF) (Immunotools, 11343663), 800 ng/ml FGF2, and 0.1 ng/ml heparin and injected subcutaneously into the abdominal tissue along the peritoneal midline into mice. Four days after the injection mice were euthanized and matrigel plugs were removed and fixed in 4% paraformaldehyde. Four animals per group were used according to G Power analysis. The statistical review was performed by a biomedical statistician.

### Immunohistochemistry (IHC)

Samples were embedded in optimal cutting temperature (OCT) compound or in paraffin and 5μm sections were cut from each sample using Thermo Shandon cryotome E. Staining with hematoxylin and eosin and IHC was performed for all samples. Staining for CD31, CD34, and αSMA was carried out using the EnVision^TM^ FLEX, High pH (Link) (Code K8000). Briefly, the sections were hydrated in ddH_2_O. Endogenous peroxidase activities were quenched in 3% H_2_O_2_ for 10 min at RT. Samples were then incubated with CD31, CD34, and αSMA accordingly, at RT for 1 h. Secondary EnVision FLEX/HRP (DAKO) was then applied for 30 min, followed by treatment with substrate/chromogen (DAKO) for 5–10 min. Slides were counterstained using Mayer’s Hematoxyl in solution. Images from at least 5 fields/section and 3 sections/implant were acquired on an Olympus BX-50 microscope.

### Statistical Analysis

Data were analyzed using SPSS 22.0 (SPSS, Inc). Continuous data were expressed as mean ± SD. Normality tests were carried out. *T*-test or Mann–Whitney test was performed accordingly (comparisons between two conditions) or ANOVA (comparisons between more than two conditions). Significant probability values were also corrected for multiple testing (Bonferroni correction). Categorical data were presented as counts and chi-square test was performed. The *P* values obtained were 2-tailed and determined to be significant at *P* ≤ 0.05.

## Results

### Differentiation of hPSCs to cSMCs and sSMCs

**FIGURE 1:**
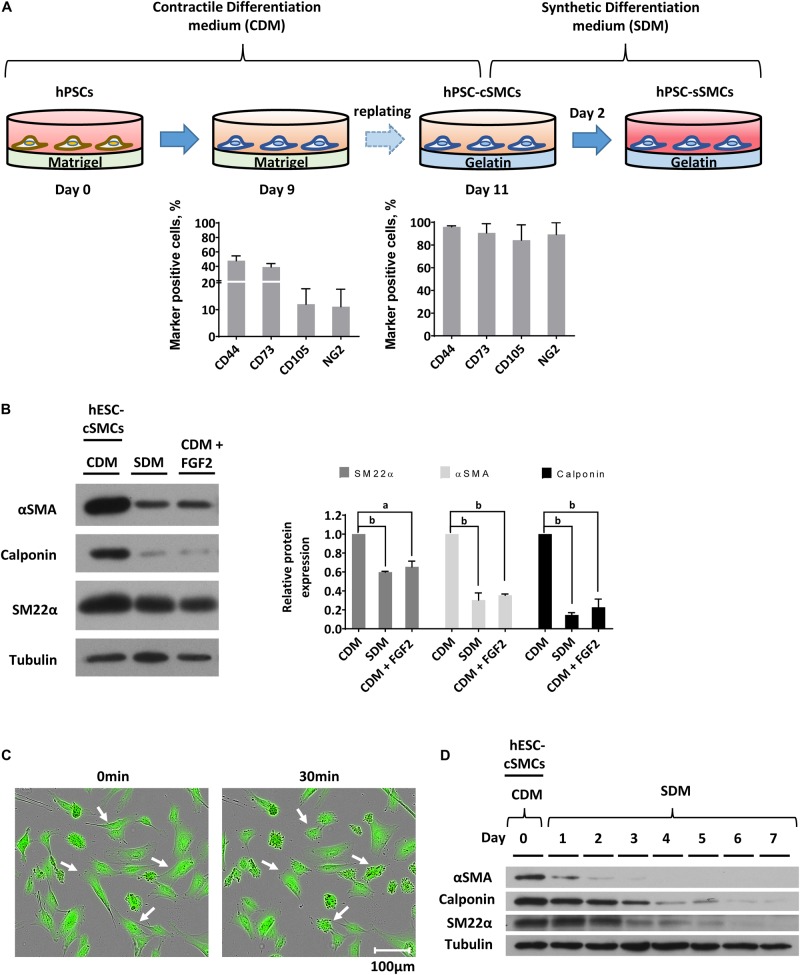
Differentiation of hPSCs to cSMCs and sSMCs. **(A)** Overview of the differentiation procedure and histograms presenting the percentage of the cells expressing the surface markers CD44, CD73, CD105, NG2 evaluated by flow cytometry analysis on day 9 (left histogram) and on day 11 (right histogram) of the differentiation process. The means ± SD were calculated from three independent experiments. **(B)** Cells on day 11 of the differentiation process (hESC-cSMCs) were further cultured in CDM, SDM, or CDM supplemented with FGF2 for 48 h. Whole-cell lysates were analyzed by SDS-PAGE and immunoblotted with antibodies recognizing αSMA, Calponin and SM22α. Quantification of band intensities for each contractile protein is presented in graphs (right). Each bar represents the intensity of the contractile protein normalized to the density of tubulin (loading control) and expressed as fold change relative to cells in CDM. The means ± SD were calculated from three independent experiments, ^a^*P* < 0.05, ^b^*P* < 0.01. **(C)** Images of hESC-cSMCs stained with calcein before (left image) and 30 min after the addition of carbachol (10^–5^M) (right image). Some contracted cells are indicated with white arrows. This is a representative image of three independent experiments. Scale bar, 100 μm. **(D)** The medium of hESC-cSMCs cultured in CDM was changed to SDM (day 0). Protein expression levels of αSMA, Calponin, SM22α from whole-cell lysates were evaluated daily until day 7 by western blot analysis. Tubulin levels served as loading control. This is a representative image of two independent experiments.

In a previous study we have generated mesenchymal stem cells (MSCs) from iPSCs by culturing them in a differentiation medium containing 10% FCs (Kouroupis et al., 2016). Since high serum is known to downregulate the expression of contractile proteins (Wanjare et al., 2013a), the key phenotypic feature of SMCs, we modified our differentiation strategy and developed a simple and quick differentiation protocol to induce the differentiation of hPSCs to cSMCs using CDM containing 2.5% FCS. After 9 days of culturing the cells in CDM, a distinct population of cells was positive for the typical mesenchymal marker CD44 (48.3 ± 0.6%) and to a lesser extent was positive for the general mesoderm marker CD73 (Boyd et al., 2009; Vodyanik et al., 2010), CD105 and NG2 (Figure 1A). Cells were then enzymatically removed, replated without sorting and cultured in CDM on gelatin coated dishes. When confluent (after 2–3 days), the cells exhibited high expression levels of the contractile proteins αSMA, Calponin, SM22α, implying a commitment to cSMCs (Figure 1B). In agreement, 78.4% ± 0.04 of the cells (from four independent experiments) contracted after treatment with carbachol (Figure 1C). Moreover, cells tested positive for typical markers expressed by MSCs as well as by MCs including CD44, CD73, CD105, CD29, and NG2 (Figure 1A). This cSMC phenotype was stable for at least 8 passages (data not shown).

**FIGURE 2:**
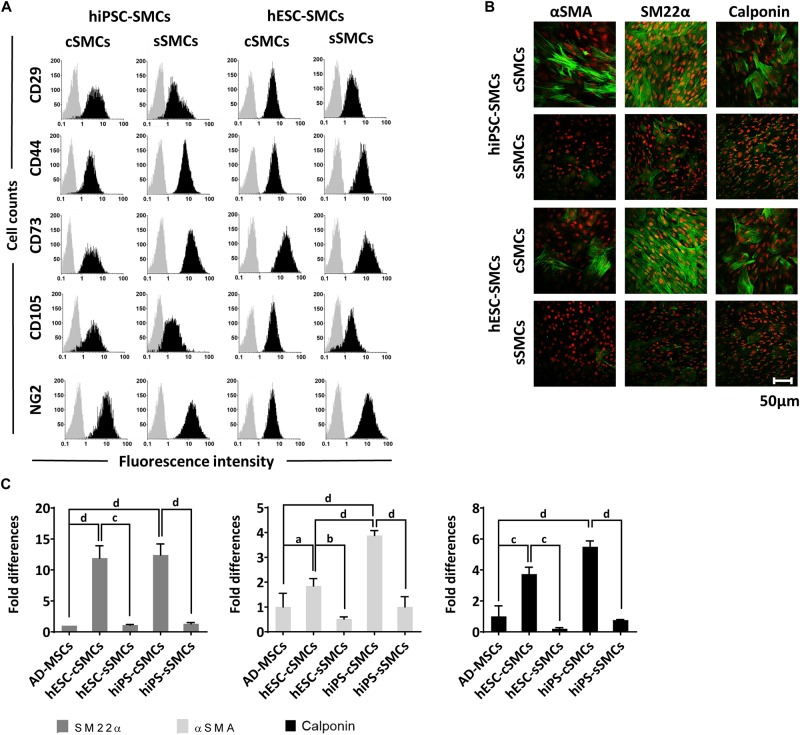
Phenotypic analysis of hPSC-cSMCs and hPSC-sSMCs. **(A)** The panel shows representative flow cytometry histograms of hiPSC-cSMCs, hiPSC-sSMCs, hESC-cSMCs, and hESC-sSMCs (columns from left to right). The histograms of the samples stained with the isotypic IgGs are shown in light gray, whereas the samples stained with fluorochrome-conjugated antibodies are overlaid in black. Each histogram is a representative of at least three independent experiments. **(B)** Immunofluorescence analysis performed on hiPSC-cSMCs, hiPSC-sSMCs, hESC-cSMCs, and hESC-sSMCs. Green fluorescence indicates cells positive for αSMA (left column), SM22α (middle column), and Calponin (right column) whereas red indicates nuclei (PI stain). Images were taken on Leica TCS SP5 confocal microscope and they are representative of three independent experiments. Scale bar, 50 μm. **(C)** Relative numbers of cells expressing SM22α, αSMA and Calponin from AD-MSCs, hESC-cSMCs, hESC-sSMCs, hiPSC-cSMCs, and hiPSC-sSMCs (histograms left, middle and right respectively) are expressed as fold differences based on quantification of the immunofluorescence analysis **(B)**. The means ± SD were calculated from three independent experiments, ^a^*P* < 0.05, ^b^*P* < 0.01, ^c^*P* < 0.001, ^d^*P* < 0.001.

In order to induce the transition of hPSC-cSMCs to hPSC-sSMCs, we added FGF2 to the differentiation medium, a growth factor known to induce the phenotypic switch from primary cSMCs to sSMCs (Jackson and Reidy, 1993). After 2 days of treatment the protein expression of the contractile proteins was downregulated, a fact consistent with the development of the synthetic phenotype (Figure 1D and Supplementary Figure S1A). Given that other growth factors have also a similar effect (Holycross et al., 1992), we treated the cells with SDM (basal medium containing 2% FCS, supplemented with a combination of growth factors including EGF, FGF2, and IGF-I) and the contractile proteins as well as their gene expression were downregulated and were almost undetectable after 3–4 days (Figure 1D and Supplementary Figures S1B,C). Upon further phenotypic characterization of hPSC-cSMCs and hPSC-sSMCs no significant change in the surface expression of the mesenchymal/MC markers CD29, CD44, CD73, CD105, NG2 was found between the two subtypes (Figure 2A). However, there were statistically significant fewer cells expressing αSMA, Calponin, SM22α in hPSC-sSMCs (similar to adipose derived-MSCs, AD-MSCs) compared to hPSC-cSMCs (Figures 2B,C). Finally, the pluripotent markers Nanog and Sox2 were undetectable from the first passage in both phenotypes (Supplementary Figure S1D).

The expression of SMMHC, a marker of cSMC maturation, was detectable, albeit at a low level, in hPSC-cSMCs indicating an immature phenotype of the cells (Wanjare et al., 2013a). We then attempted to induce maturation by exposure of differentiated hESC-cSMCs to TGFβ1/heparin. Indeed, after 24 h of treatment a significantly upregulated gene expression of the contractile protein SMMHC (ninefold induction) was observed (Supplementary Figure S2A). The protein expression of the other contractile proteins was increased but did not reach statistical significance (Supplementary Figure S2B). Notably, the proliferation potential of the hESC-cSMCs TGFβ1/heparin treated cells was decreased compared to hESC-cSMCs (Supplementary Figure S2C), all consistent with the acquisition of a mature phenotype (Wanjare et al., 2013a).

**FIGURE 3:**
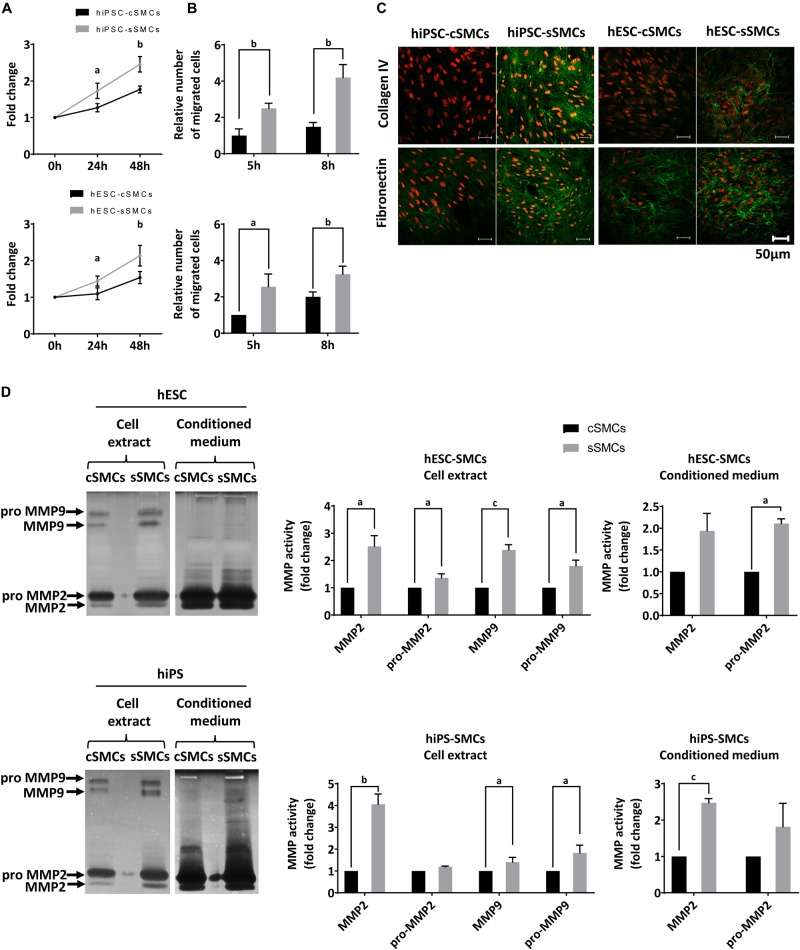
Functional analysis of hPSC-cSMCs and hPSC-sSMCs. **(A)** Proliferation assay. Numbers of hiPSC-cSMCs, hiPSC-sSMCs (upper graph) and hESC-cSMCs, hESC-sSMCs (lower graph) are expressed as fold change at 24 and 48 h relative to 0 h and presented as means ± SD from three independent experiments. ^a^*P* < 0.05, ^b^*P* < 0.01. **(B)** Migration assay. Relative number of migrated hiPSC-cSMCs, hiPSC-sSMCs (upper chart) and hESC-sSMCs, hESC-cSMCs (lower chart) at 5 and 8 h using the wound healing assay are presented as means ± SD from three independent experiments. ^a^*P* < 0.05, ^b^*P* < 0.01. **(C)** Extracellular matrix deposition. Immunofluorescence analysis performed on hiPSC-cSMCs, hiPSC-sSMCs, hESC-cSMCs, and hESC-sSMCs. Green fluorescence indicates Collagen IV (upper panel) and Fibronectin (lower panel), whereas red fluorescence indicates nuclei (PI stain). Images were taken on Leica TCS SP5 confocal microscope and are representative of three independent experiments. Scale bar, 50 μm. **(D)** MMP2 and 9 activity. hESC-cSMC, hESC-sSMC (upper image) and hiPSC-cSMCs, hiPSC-sSMCs (lower image) cell extracts and conditioned media were assessed using gelatin zymography. The images are inverted photos of the zymograms and are representative of three independent experiments. Relative MMP activities were evaluated by quantification of band intensities and are presented in the charts. Data are expressed as means ± SD from three independent experiments. ^a^*P* < 0.05, ^b^*P* < 0.01, ^c^*P* < 0.001.

Functional characterization of these two cell subtypes (hPSC-cSMCs and hPSC-sSMCs) revealed that hPSC-sSMCs exhibited higher proliferation and migration potential (Figures 3A,B) and increased deposition of ECM (fibronectin, collagen IV) (Figure 3C), compared to hPSC-cSMCs. Moreover, increased MMP-2 activity both in the conditioned medium and in the cell extract and MMP-9 activity in the cell extract was found in hPSC-sSMCs compared to hPSC-cSMCs (Figure 3D). All of the above results are consistent with the phenotypes of primary sSMCs and cSMCs (Beamish et al., 2010; Cecchettini et al., 2011).

Finally, both cell subtypes demonstrated similar potential to differentiate toward chondrogenic and osteogenic lineages (Supplementary Figure S3A).

### MC Functionality of hPSC-cSMCs and hPSC-sSMCs

**FIGURE 4:**
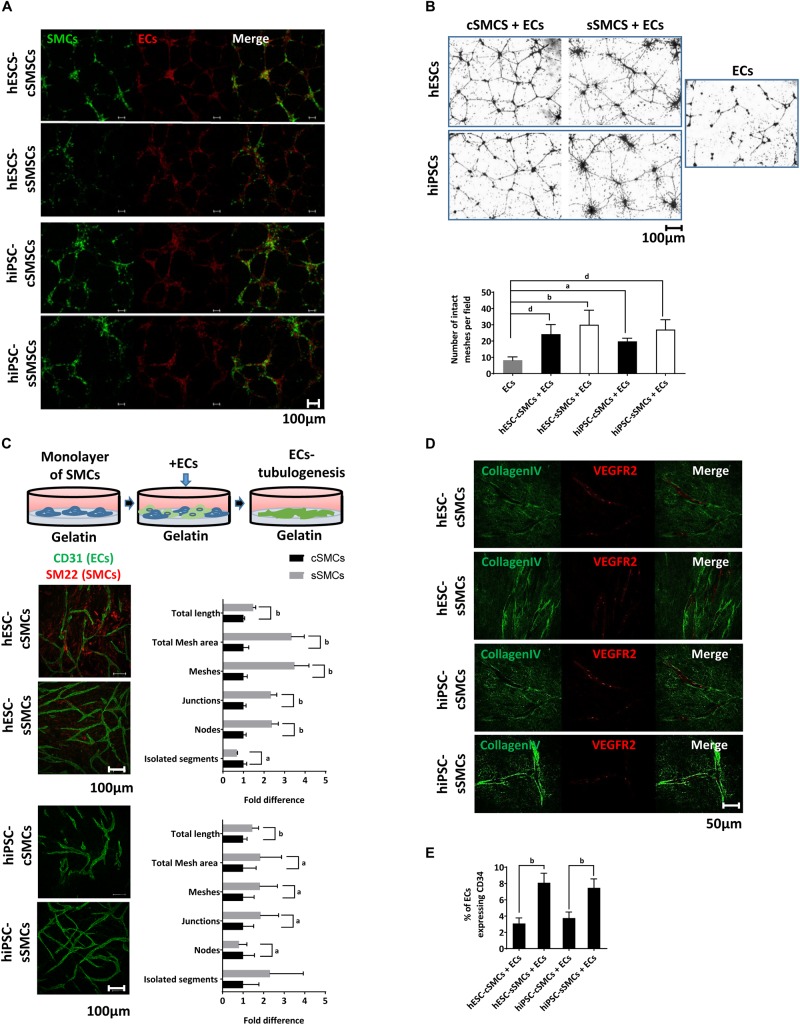
MC function of hPSC-cSMCs and hPSC-sSMCs. **(A)** *In vitro* angiogenesis assay. hESC-cSMCs, hESC-sSMCs, hiPSC-cSMCs, and hiPSC-sSMCs labeled with PKH67, shown in green, were mixed in a ratio 1:9 with primary ECs labeled with PKH26, shown in red, and allowed to generate a vascular network on matrigel for 8 h. Merged fluorescence images are shown in right column of each panel. Images were taken on Leica TCS SP5 confocal microscope and are representative of three independent experiments. Scale bar, 100 μm. **(B)** Regression analysis. Phase-contrast images of hESC-cSMCs + ECs, hESC-sSMCs + ECs (upper left panel), hiPSC-cSMCs + ECs, hiPSC-sSMCs + ECs (lower left panel) and ECs (right panel) derived vascular networks on matrigel at 48 h are shown. (Images were taken on Zeiss axiovert 100, and are representative of three independent experiments. Number of intact meshes representing the vascular network integrity for each condition are shown in the chart. Graph data are expressed as means ± SD from three independent experiments. ^a^*P* < 0.05, ^b^*P* < 0.01, ^d^*P* < 0.001. Scale bar, 100 μm. **(C)** Tubulogenesis assay. ECs were plated on a monolayer of hESC-cSMCs, hESC-sSMCs (upper images) or hiPSC-cSMCs, hiPSC-sSMCs (lower images) and allowed to generate tubule-like structures (illustration). Green fluorescence indicates ECs (CD31 staining). Images were taken on Leica TCS SP5 confocal microscope and are representative of three independent experiments. Morphometric analysis of the hESC-cSMCs + ECs, hESC-sSMCs + ECs and hiPSC-cSMCs + ECs, hiPSC-sSMCs + ECs tube-like structures is presented in the charts and expressed as fold change between the two conditions regarding various parameters. Analysis was performed with ImageJ software. Graph data are expressed as means ± SD from three independent experiments. ^a^*P* < 0.05, ^b^*P* < 0.01. Scale bar, 100 μm. **(D)** Immunofluoresence analysis of the hESC-cSMCs + ECs, hESC-sSMCs + ECs and hiPSC-cSMCs + ECs, hiPSC-sSMCs + ECs tube-like structures was performed. Green fluorescence indicates Collagen IV expression (left column) whereas red fluorescence indicates ECs (VEGFR2 staining) and the merge is shown in the right column. Images were taken on Leica TCS SP5 confocal microscope and are representative of three independent experiments. Scale bar, 100 μm. **(E)** The percentage of ECs expressing CD34 in hESC-cSMCs + ECs, hESC-sSMCs + ECs, hiPSC-cSMCs + ECs and hiPSC-sSMCs + ECs tube-like structures is presented in the chart. CD34 positive ECs were evaluated after cell detachment, double-staining with anti-CD31-FITC, anti-CD34-PE antibodies and flow cytometry analysis. The percentage of CD34 + /CD31 + cells ratio is expressed in the chart as means ± SD from three independent experiments. ^b^*P* < 0.01.)

To explore whether our SMC derivatives could assist in vessel formation by ECs and stabilization of the neovasculature, both cell subtypes were co-cultured with primary human ECs on matrigel matrix (*in vitro* angiogenesis assay). A fixed ratio of 9:1 ECs:SMCs (hPSC-cSMCs or hPSC-sSMCs) similar to the average ratio of MC:ECs found in vascular beds *in vivo* was used (Shepro and Morel, 1993). As shown in Figure 4A both hPSC-cSMCs and hPSC-sSMCs integrated into the ECs network. Furthermore, no statistically significant difference could be detected between hESC-cSMCs + ECs, hESC-sSMCs + ECs and ECs alone regarding morphometric parameters of the capillary-like structures quantified by ImageJ software (Supplementary Table S1). Moreover, both cell subtypes stabilized the vascular network and inhibited its regression, a key feature of MCs (Figure 4B). On the contrary, AD-MSCs or hiPSC-MSCs (Kouroupis et al., 2016) used in the same experimental set up could not rescue the ECs network from regression (Supplementary Figure S3B), implying that hPSC-cSMCs and hPSC-sSMCs, although exhibiting some features of MSCs, are functional MCs. We next explored the ability of hPSC-cSMCs and hPSC-sSMCs monolayers to induce ECs tubulogenesis. When ECs were added on top of a hPSC-sSMC monolayer, they were organized into a significantly more complex network, as evaluated with various morphometric parameters, compared to that developed by ECs added on a hPSC-cSMC monolayer (Figure 4C). Moreover, immunostaining of collagen IV (a component of vascular basement membrane) in the hPSC-sSMC + EC co-culture clearly demarcated the generated vascular tubules indicating a more mature network compared to hPSC-cSMC + ECs, where a less continuous and lower staining intensity was observed (Figure 4D). No significant difference in EC or SMC number was found between the two conditions that could account for this effect (data not shown). Furthermore, more CD34 + ECs, indicating more tip cells (Siemerink et al., 2012) and therefore higher angiogenic potential, were found in the hPSC-sSMC + EC co-cultures compared to hPSC-cSMC + EC co-cultures (Figure 4E).

### Generation of Vascular Organoids: Characterization and *in vitro* Functionality

In order to maintain the stability of the phenotype of hPSC derived SMC subtypes and also maximize their MC function by resembling the 3D native tissue environment, we generated a 3D spheroidal co-culture model with human ECs.

**FIGURE 5:**
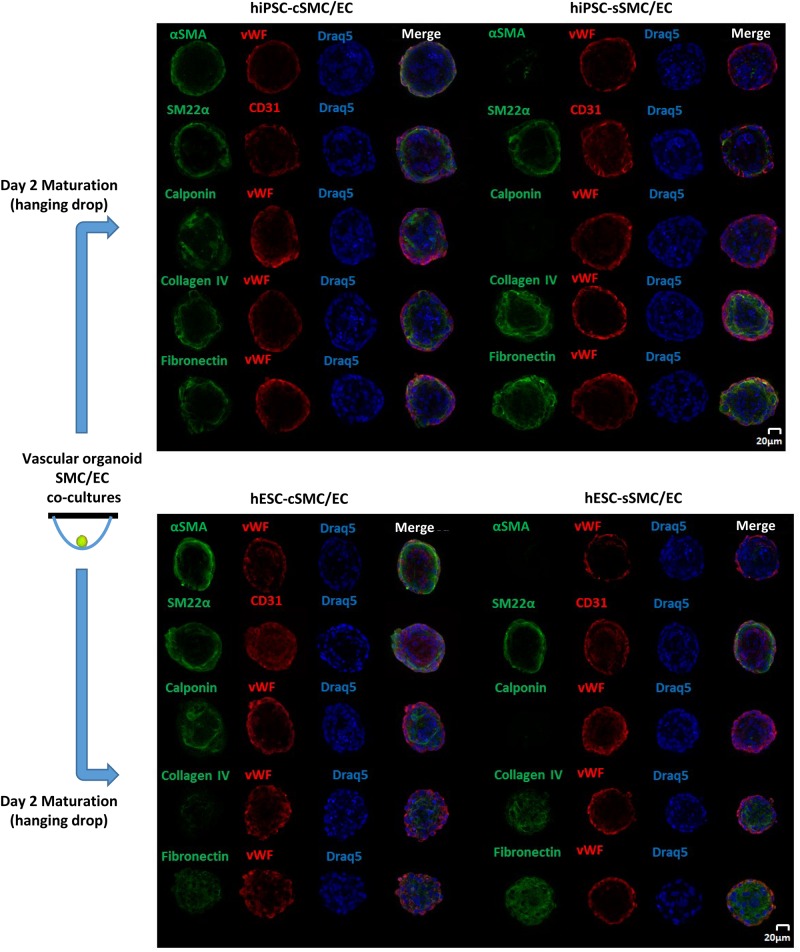
Generation and Immunophenotypic characterization of vascular organoids. Vascular organoids were generated in 2 days using the hanging drop method (illustration). Immunofluoresence analysis was performed on vascular organoids consisting of hiPSC-cSMC/EC, hiPSC-sSMC/EC **(upper panel)** and hESC-cSMC/EC, hESC-sSMC/EC **(lower panel)**. Green fluorescence indicates either SMCs expressing αSMA (1st line), SM22α (2nd line), Calponin (3rd line), Collagen IV expression (4th line), and Fibronectin expression (5th line) whereas red indicates ECs (von Willebrand or CD31 staining) and blue indicate nuclei (Draq5 staining). Images were taken on Leica TCS SP5 confocal microscope and are representative of at least three independent experiments. Scale bar, 20 μm.

First, we analyzed self-organization of 1,000 hPSC-SMC/ECs into one vascular organoid, when co-cultured in EGM2 medium/methylcellulose using the hanging drop method. SMCs (hPSC-cSMCs or hPSC-sMCs):ECs, at a 1:9 cell ratio, co-cultures aggregated rapidly and condensed into 3D vascular organoids within 48 h. Using pre-labeled cells, as well as indirect immunofluorescence, we observed that ECs were distributed on the surface of the vascular organoids whereas SMCs were located underneath the ECs (Figure 5). Immunofluoresence analysis of the cells revealed that hPSC-cSMCs highly expressed contractile proteins compared to hPSC-sSMCs, indicating a phenotype identical to that observed, when the cells were cultured in monolayers (Figure 5). In the same context, hPSC-sSMC/EC vascular organoids were characterized by increased deposition of ECM compared to hPSC-cSMC/EC vascular organoids (Figure 5).

**FIGURE 6:**
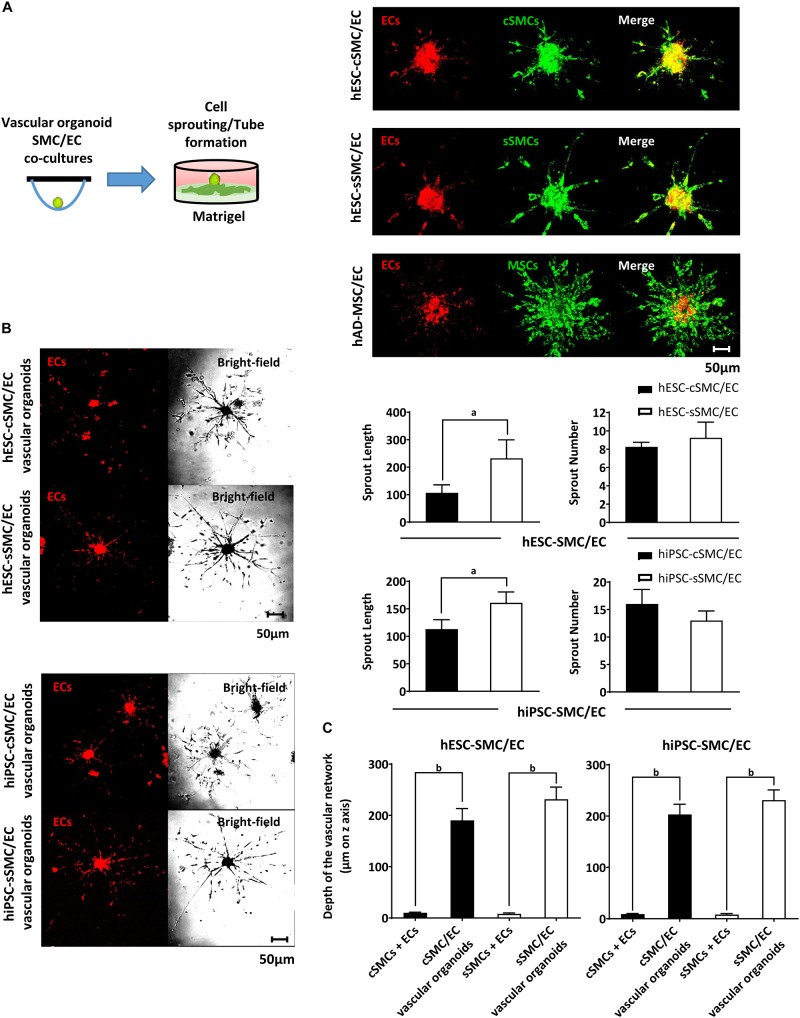
Functional characterization of vascular organoids *in vitro.* **(A)** hPSC-SMC subtype/EC vascular organoids generated using the hanging drop method, were added on matrigel and allowed to develop sprouts (illustration). Representative images of hESC-cSMC/EC, hESC-sSMC/EC vascular organoids and hAD-MSC/EC spheroids 3d after their addition to matrigel. Red immunofluorescence indicates ECs (prelabeled with PKH26, left column), green fluorescence indicates hESC-cSMCs, hESC-sSMCs, and hAD-MSCs (pre-labeled with PKH67, middle column) and the merge is shown in the right column. Images were taken on Leica TCS SP5 confocal microscope and are representative of at least three independent experiments. Scale bar, 50 μm. **(B)** Representative images of (hESC-cSMC/EC, hESC-sSMC/EC vascular organoids (upper panel) and hiPSC-cSMC/EC, hiPSC-sSMC/EC vascular organoids (lower panel) 3d after their addition on matrigel. Red immunofluorescence indicates ECs (prelabeled with PKH26, left column), whereas phase-contrast images of the same microscopic field are shown on the right column. Images were taken on Leica TCS SP5 confocal microscope and are representative of at least three independent experiments. Scale bar, 50 μm. Charts presenting length of sprouts (left) and number of sprouts (right) from hESC-cSMC/EC, hESC-sSMC/EC vascular organoids (upper charts) and hiPSC-cSMC/EC, hiPSC-sSMC/EC vascular organoids (lower charts). Images were taken 3d after the vascular organoids were plated on matrigel. Number and length of the sprouts were quantified using imageJ software and they are expressed as means ± SD from three independent experiments. ^a^*P* < 0.05. **(C)** Charts presenting the depth of the vascular network from hESC-cSMCs + ECs, hESC-sSMCs + ECs single cells or equal number of cells organized as vascular organoids (left chart) and hiPSC-cSMCs/ECs, hiPSC-sSMCs/ECs single cells or equal number of cells organized as vascular organoids (right chart). Images were taken 3d after single cells or vascular organoids had been plated on matrigel. Distance of vascular network invasion in the matrigel was quantified on Leica TCS SP5 confocal microscope (z axis) and it is expressed as mean ± SD from three independent experiments. ^b^*P* < 0.01.)

In addition to phenotypical characterization, we tested the angiogenic potential of hPSC-SMC/EC vascular organoids in the matrigel sprouting assay *in vitro*. When vascular organoids were placed on matrigel, sprouts originated from them and were organized into a capillary-like network (Figure 6A), which was histomorphologically intact for longer period of time compared to the network generated by monocells (data not shown). Interestingly, unlike AD-MSC/EC mixed spheroids, at hPSC-SMC/EC vascular organoids both hESC–SMCs subtypes and ECs co-assembled in the sprouts (Figures 6A,B). Morphometric analysis of the sprouts originating from the vascular organoids revealed significantly longer sprouts originating from hPSC-sSMC/ECs compared to hPSC-cSMC/ECs (Figure 6B). Moreover, the network derived from hPSC-SMC/EC vascular organoids invaded into the matrigel, whereas the network generated by hPSC-SMCs + ECs monocells, remained on the surface of the matrigel (Figure 6C).

**FIGURE 7:**
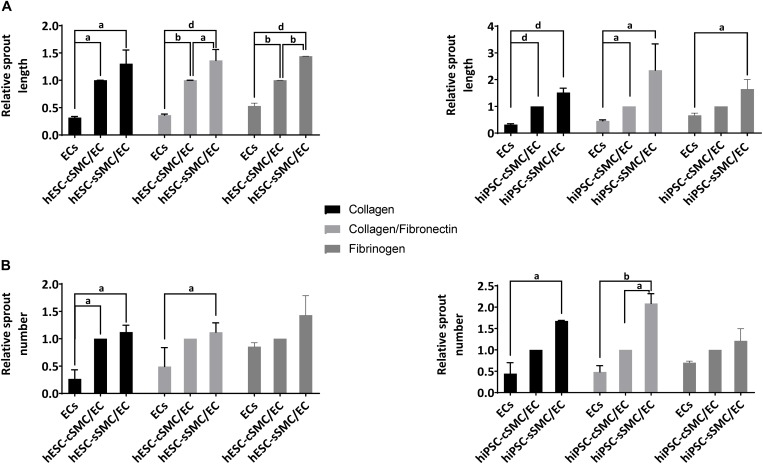
Functional characterization of vascular organoids on hydrogels composed of defined ECM components. hPSC-SMC subtype/EC vascular organoids were added on hydrogels composed of collagen, collagen/fibronectin or fibrinogen and allowed to develop sprouts for 24 h. Length and number of sprouts were quantified using imageJ software. **(A)** Relative sprout length and **(B)** relative number of sprouts between EC spheroids and hPSC-cSMC/EC, hPSC-sSMC/EC vascular organoids from three independent experiments are presented in the charts. ^a^*P* < 0.05, ^b^*P* < 0.01, ^d^*P* < 0.0001.

In order to explore further the angiogenic potential of the hPSC-SMC/EC vascular organoids in a more defined matrix, which, unlike matrigel, is devoid any incorporated growth factors, we generated hydrogels consisting of ECM components, such as collagen I, fibronectin and fibrinogen. As shown in Figure 7, hPSC-cSMC/EC and hPSC-sSMC/EC vascular organoids exhibited a sprouting profile similar to matrigel. Interestingly, hPSC-SMCs and ECs as monocells failed to generate any network, when seeded on these matrices (data not shown).

### *In vivo* Functionality of Vascular Organoids/Spheroids

**FIGURE 8:**
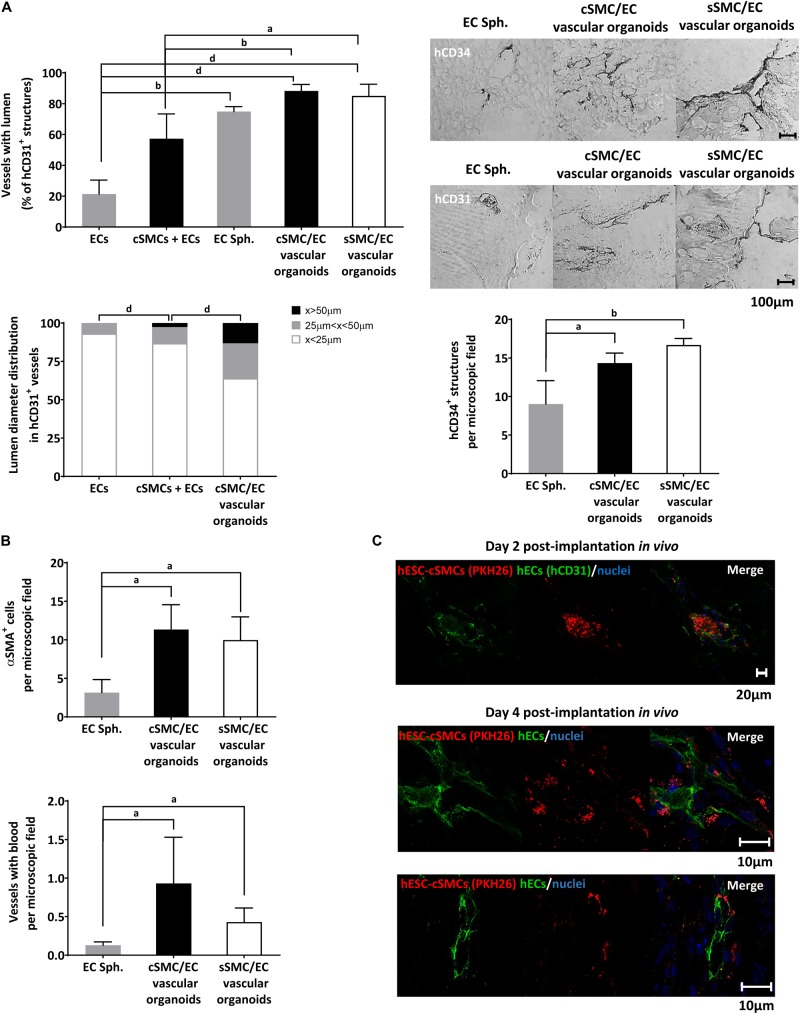
Functional characterization of vascular organoids *in vivo.* **(A)** Chart presenting percentage of vessels of human origin (anti-hCD31 positive) with lumens in ECs, hESC-cSMCs + ECs single cell matrigel implants and in EC spheroids, hESC-cSMC/EC, hESC-sSMC/EC vascular organoid matrigel implants, 4 days after the implantation (upper). Vascular structures of human origin (anti-hCD31 positive) with lumen were quantified per microscopic field and expressed as percentage of the total anti-hCD31 positive structures. Chart presenting the lumen distribution of human vascular structures (anti-hCD31 positive) in ECs, hESC-cSMC/EC single cell matrigel implants and in hESC-cSMC/EC vascular organoid matrigel implants, 4 days after the implantation (left). Chart presenting the number of anti-hCD34 (positive structures in EC spheroid, hESC-cSMC/EC and hESC-sSMC/EC vascular organoid matrigel implants, 4 days after the implantation (right). All quantifications were performed in at least five fields and three sections per implant. All graph data are expressed as means ± SD from four animals per condition. ^a^*P* < 0.05, ^b^*P* < 0.01, ^d^*P* < 0.001. Representative images of sections stained with anti-hCD34 antibody (upper panel) or anti-hCD31 antibody (lower panel) using immunohistochemistry in EC spheroids, hESC-cSMC/EC, and hESC-sSMC/EC vascular organoid matrigel implants (images from left to right), 4 days after the implantation. Scale bar, 100 μm. **(B)** *In vivo* analysis of vessel maturation and perfusion. Sections from EC spheroids, hESC-cSMC/EC and hESC-sSMC/EC vascular organoid matrigel implants 4 days after implantations were stained with anti-αSMA antibody and the number of positive cells per microscopic field was quantified and presented in the upper chart. Vascular structures containing red blood cells/microscopic field in EC spheroid, hESC-cSMC/EC and hESC-sSMC/EC vascular organoid matrigel implants 4 days after implantation are presented in the lower histogram. All quantifications were performed in at least five fields and three sections per implant. All graph data are expressed as means ± SD from four animals per condition. ^a^*P* < 0.05. **(C)** Immunofluoresence analysis of sections from ECs spheroids, hESC-cSMC/EC and hESC-sSMC/EC vascular organoid matrigel implants 2 days (upper panel) and 4 days (lower panels) after implantation. Green fluorescence indicates human ECs (anti-hCD31-FITC staining) (left images), red fluorescence indicates hESC-cSMCs or hESC-sSMCs (prelabeled with PKH26) and blue fluorescence indicates nuclei (Draq5 staining). Merged images are shown (right images). Images were taken on Leica TCS SP5 confocal microscope and are representative of 2 animals (2 days) and 4 animals (4 days). Scale bars, 10, 20 μm.)

To compare *in vivo* functionality of the differentiated cells, we employed a matrigel plug assay using our hESC–SMCs subtypes mixed with primary human ECs as monocells. After 4 days of subcutaneous transplantation, more vessel structures of human origin with lumen and a broader distribution of vascular diameter were found in the vascular networks of dual cell implants (EC and hESC-cSMC) compared and to sole EC implants (Figure 8A). hESC-SMCs, however, could not induce capillary growth, when they were implanted in matrigel plugs without ECs (data not shown).

We then wanted to test our hypothesis that hESC-SMC (cSMC or sSMC)/EC vascular organoids, when implanted in a matrigel plug, would lead to an earlier maturation and stabilization of the vascular network compared to sole EC spheroid implants. Analysis of the vascular structures derived from human ECs in implants harvested from mice as early as 4 days after implantation, revealed significantly more luminal vessels in spheroid implants (vascular organoids or EC spheroids) compared to mono-EC implants (Figure 7A). Moreover, higher number of human vessels with lumen were found in hESC-SMC (cSMC and sSMC)/EC vascular organoid implants compared to hESC-cSMCs + ECs monocell implants.

Further analysis with respect to vessel diameter and classification according to their size revealed that more hCD31 positive vascular luminal structures and with greater lumen diameter were found in the hESC-cSMC/EC vascular organoid implants compared to mixed or sole EC monocell implants (Figure 8A). Statistically significant more vascular structures of human origin expressing CD34 (hCD34+) were observed in hESC-SMC (cSMC and sSMC)/EC vascular organoids compared to EC spheroid implants (Figures 8A,B). hESC-sSMC/EC vascular organoid implants had also more hCD34 positive structures compared to hESC-cSMC/EC vascular organoid implants, a result which is in agreement with the increased expression of CD34 in ECs, when co-cultured with hESC-sSMC (Figure 4E). Moreover, human vasculature that emerged from implanted hESC-cSMC/EC and hESC-sSMC/EC vascular organoids formed more anastomoses with mouse vasculature and more perfused blood vessels compared to EC spheroids (Figure 8B).

As shown in Figure 8B, in implants containing hESC-cSMC/EC and hESC-sSMC/EC vascular organoids, significantly more blood vessels were covered with αSMA-positive cells, compared to EC spheroid implants, indicating that vessels were probably stabilized by hESC-cSMC and hESC-sSMC.

To test the origin of the mural layer of the newly formed vessels, experiments were conducted in which red labeled hESC-cSMC/hESC-sSMC in vascular organoids with EC were implanted into mice. At day 2 following implantation, ECs started assembling into tubes originating from the vascular organoids and immunofluoresence analysis revealed red labeled cSMCs in close proximity to ECs tubes (green hCD31 staining), indicating human donor origin of the MCs of the assembled vessels (Figure 8C). Indeed, 4 days post-implantation red labeled hESC-cSMCs and hESCs-sSMCs where found next to human vascular structures (Figure 8C). The possibility however that murine PCs or SMCs were also recruited from surrounding mouse tissue cannot be excluded. Overall, these results show that EC spheroids displayed lower ability to form stable and perfused vascular networks as early as in 4 days compared to vascular organoids containing either SMC subtype.

## Discussion

In the current study, we developed a quick and robust method to generate both cSMCs and sSMCs from hPSCs. Moreover, the assembly of these SMC subtypes into 3D spheroid co-cultures served as focal points for the sprouting of capillary-like structures *in vitro* and their delivery *in vivo* led to rapid generation of a complex functional vascular network.

In our previous study (Kouroupis et al., 2016) hiPSCs were fully committed to the mesodermal lineage by gradually increasing the serum concentration to 10% for 20 days *in vitro*. Since serum downregulates the expression of contractile proteins (Wanjare et al., 2013b), using a short-term (11 days) reduced-serum induction protocol, we have successfully induced hPSCs toward the cSMC phenotype (spindle-shaped morphology, >90–95% positivity for NG2, and CD29, CD44, CD73, CD105 mesodermal markers, high expression of early contractile markers αSMA, Calponin, and SM22α, low expression of the maturation marker SMMHC),which contracted when exposed to carbachol (Owens et al., 2004; Rensen et al., 2007). Kumar et al. (2017) have also generated cSMCs from hiPSCs using a longer differentiation protocol through a mesenchymoangioblast intermediate population, by exposure of cells first to high serum-containing media (Wanjare et al., 2013a), followed by low-serum induction. According to our differentiation protocol, a combination of low serum conditions and subsequent seeding of the cells on gelatin, as ECM coating, was adequate to rapidly generate an immature cSMC phenotype (αSMA^high^, Calponin^high^, SM22^high^, SMMHC^+^) that remained stable and without signs of senescence for at least 8 passages. Furthermore, short treatment with TGFβ1 (Chen et al., 2016) and heparin (Hashimoto et al., 2005) induced maturation of the hPSC-cSMCs seen by the upregulated expression of SMMHC, the most mature marker that demarcates the contractile vSMCs (Wanjare et al., 2013a). Finally, we induced transition of hPSC-cSMC to sSMCs (hPSC-sSMC), a phenotype present during neovascularization, embryonic vessel development and in injured or diseased vessels during vessel remodeling (Wanjare et al., 2013b), by the addition of FGF2, a mitogen known to promote primary vascular cSMC conversion to sSMCs (Jackson and Reidy, 1993). Indeed, short-term FGF2 exposure (2 days) resulted in downregulation of αSMA, Calponin, and SM22α expression, consistent with the development of hPSC-sSMCs. In addition, hPSC-sSMCs exhibited characteristic functions of sSMCs (Hao et al., 2003; Rensen et al., 2007), such as increased cell proliferation and migration, ECM protein deposition and upregulated MMP-2 and 9 activity compared to hPSC-cSMCs.

Both hPSC-cSMCs and sSMCs exhibited similar multipotent potential giving rise to osteocytes and chondrocytes, a typical feature of mesenchymal precursors which has been also described in MCs (Crisan et al., 2008; Speer et al., 2009; Majesky et al., 2017). In fact, it has been shown that some adult multipotent stromal cells might belong to a subset of MCs (Crisan et al., 2008). Indeed, *in vitro* tri-lineage differentiation of PCs has been documented (Farrington-Rock et al., 2004; Crisan et al., 2008), however this has been questioned regarding SMCs (Nguyen et al., 2013; Tang et al., 2013). Using fate mapping and lineage tracing approaches the multilineage potential for osteogenic and chondrogenic differentiation of SMCs has been attributed either to a reprogramming-like process of differentiated SMCs that leads to the generation of multipotent progenitor cells (Speer et al., 2009; Majesky et al., 2017) or to the presence of a progenitor cell population, such as multipotent vascular stem cells-MVSCs in the SMC culture that gives rise to both SMCs subtypes and to other lineages as well (Tang et al., 2012) or to another mesenchymal precursor (Kumar et al., 2017).}. The lack of SMC culture homogeneity at a specific differentiation stage, which may include immature and partially differentiated MVSCs, and also the specificity of the marker used for the lineage tracing might explain the disparity of the various reports (Nguyen et al., 2013; Tang et al., 2013). Based on these studies, our hPSC-cSMCs having a rather immature phenotype (proliferation potential, low expression of SM MHC) could exhibit multilineage potential as well. However, the existence of a minor partially differentiated subpopulation in the hPSC-SMCs culture cannot be excluded.

MCs are committed to stabilize the vasculature by paracrine and cell-cell interactions with neighboring ECs (Armulik et al., 2011). However, characterization of cSMCs and sSMCs derived from hPSCs regarding their MC function is limited in the literature. Our study reveals that both cell subtypes integrate in the EC network on matrigel and inhibit its regression, two essential features of MCs and important aspects for the use of these cells in tissue engineering applications. AD-MSCs as well as hiPSC-MSCs (Kouroupis et al., 2016) failed to inhibit the EC network regression indicating that our generated cells, although exhibiting some phenotypic and functional features of MSCs, display mainly MC functions. Furthermore, both cSMCs and sSMCs monolayers induced EC tubulogenesis. Notably, sSMCs stimulated the organization of a more complex and expanded EC network compared to cSMCs as indicated by morphometric parameters, generation of a vascular basement membrane structure and higher percentage of ECs expressing CD34, a marker of tip cells (Siemerink et al., 2012). This differential effect of the two subtypes could be either due to specific SMC subtype-EC interactions or to increased extracellular deposition of fibronectin and collagen IV by sSMCs that could act as a provisional matrix promoting the EC tubulogenesis. These results are in agreement with the physiological role of sSMCs during both neovascularization in the embryo and vascular remodeling in adult blood vessels (Wanjare et al., 2013b).

In line with these findings, the generated SMC derivatives could demonstrate an important building block toward the study of developmental processes and diseases implicating these cell subtypes. In addition, they could also be used for the reconstruction of physiologically relevant vasculature. Specifically, the use of a rather immature but committed hPSC-cSMC, exhibiting both proliferation (unlike mature cSMCs) and maturation potential, might be favorable for tissue engineering applications. However, transplantation of mono-cells *in vivo* is usually characterized by low survival rate (Hayashi et al., 2004). Moreover, the lack of SMC culture homogeneity at a specific differentiation stage, the SMC phenotypic plasticity as well as the fact that SMC phenotype and function are tightly regulated by their surrounding microenvironment and by their organization within the tissue (Rensen et al., 2007), all indicate a high risk of SMCs to acquire an unfavorable (inflammatory) phenotype, when implanted as monocells in a hostile environment (Chistiakov et al., 2015). These obstacles could be overcome by the generation of 3D cell structures (small-scale vascular organoids) containing both ECs and SMCs subtypes, which unlike traditional 2D monolayer cultures, would provide enhanced cell–cell interactions that closely mimic the natural/physiological tissue microenvironment with beneficial effects on cell survival, phenotypic stability and function, when transplanted *in vivo* (Derda et al., 2009; Bhang et al., 2011). In Korff and Augustin (1998) introduced 3D EC spheroids as an *in vitro* model exhibiting angiogenic responses and sprouting behavior *in vivo*. Since then, multi-cellular spheroids have become a common 3D cell culture system, generated either from one or many cell types for multiple applications (reviewed in Laschke and Menger, 2017). Accordingly, we efficiently generated for the first time 3D SMC-EC vascular organoids using both hPSC-SMC subtypes (cSMCs and sSMCs) and ECs. Randomly mixed hPSC-cSMCs:ECs or hPSC-sSMCs:ECs in a fixed ratio of 1:9 underwent self-assembly into a segregated 3D structure similar to primary vSMC/EC spheroids (Korff et al., 2001) representing the physiological assembly of a normal blood vessel. Specifically, they were characterized by a multicellular spheroidal SMC core and an outer EC layer, which can be regarded as an inside-out assembly of a resting vessel wall. Phenotypic analysis of hPSC-SMCs, when co-cultured with ECs in vascular organoids, showed preservation of the two subtype signatures, since coalescence of hPSC-cSMCs/ECs were characterized by high expression of contractile proteins (Calponin, αSMA and SM22α) whereas hPSC-SMC/EC 3D vascular organoids significantly enhanced the deposition of ECM proteins, such as fibronectin and collagen IV. Implantation of the generated vascular organoids in matrigel or hydrogels composed of individual ECM components led to increased capillary network sprouting, which was characterized by SMC-EC co-alignment within the generated sprouts. However, since MCs are characterized by high plasticity involving a continuum of cell phenotypes, from PCs to SMCs, including transitional cell phenotypes as well (Holm et al., 2018), the acquisition of a phenotype closer to PCs by the SMC derivatives in the generated capillary-like network cannot be excluded. hPSC-sSMC/EC vascular organoids gave rise to longer sprouts compared to hPSC-cSMC/EC probably due to the migratory profile and higher MMP2 activity (and subsequent ECM degradation) of hPSC-sSMC, both associated with vessel remodeling. hPSC-SMC subtype/EC vascular organoids were superior to EC spheroids regarding sprouting, while hPSC-SMC and EC monocells failed to generate a capillary like network in simple ECM hydrogels. In summary, the generated SMC-EC vascular organoids preserved the phenotypic and functional signatures of the two SMCs subtypes, and exhibited the potential to give rise to a durable (compared to mono-cells) 3D vascular network *in vitro*.

Upon subcutaneous implantation in mice, hESC-SMC/EC vascular organoids not only generated more lumenized vascular structures and with greater diameter compared to ECs and hESC-SMCs/ECs monocell implants, but also formed more hCD31 + /CD34 + vessels, more anastomoses with the recipient’s vasculature and more perfused vessels compared to EC spheroids. Therefore, the matrigel plug assay strongly demonstrated the capacity of both hESC-cSMC/EC and hESC-sSMC/EC vascular organoids to serve as focal starting points of outgrowing capillary sprouts consisting of both SMCs and ECs in order to generate, in a short time frame, mature human vascular structures with the ability to anastomose with resident vasculature *in vivo*. However, although we observed pre-stained hPSC-SMCs aligned along the vessels from the human ECs, we cannot exclude the participation of MCs from the host as well. Given that SMC subtypes were unable to induce neovascularization, when implanted alone (without ECs) *in vivo* as monocells, it seems that their role in the 3D vascular organoids is mainly on vascular remodeling and stabilization. Furthermore, an additional positive effect of hESC-SMC on hEC survival (Korff et al., 2001) could account for the increased number of vascular structures of human origin in the vascular organoid implants compared to EC spheroid implants. Our 3D spheroid approach is also flexible and versatile enough to enable further modifications (reviewed in Kouroupis et al., 2018), various applications regarding specific disease models, routes of administration (injectable), types of scaffolds, length of observation, which might further uncover the differential effect of hPSC-cSMC and hPSC-sSMC on vascular remodeling *in vivo*.

In summary, we developed a rapid differentiation protocol for hPSCs toward immature cSMCs, which can further mature after a short TGFβ1/heparin treatment or be induced to sSMCs after a short induction with FGF2. The phenotypic modulation of vascular SMCs is an important vascular injury repair mechanism and therefore, it plays a major role in the pathogenesis of a number of diseases, including atherosclerosis, restenosis and transplant vasculopathy (Beamish et al., 2010; Chistiakov et al., 2015). Our innovative differentiation strategy, unlike the previously reported protocols, offers the possibility of studying, in the same simple experimental set up, the molecular mechanisms underlying phenotypic plasticity of the generated hPSC-SMCs. Given that FGF-TGFβ signaling antagonism is reported as the primary regulator of the SMC phenotypes, our protocol is an ideal model to study this mechanism.

Based on the recent perception that MCs consist of a phenotypic continuing spectrum with PCs at the one end and SMCs on the other, it seems that using our differentiation protocol we generated MCs whose features cluster in the SMC phenotypic area, without excluding the existence of transitional cell types. Heterogeneity and spatiotemporal variation in protein expression is characteristic of MCs and therefore, it must be taken into consideration, when tissue engineering approaches are designed, where preservation or induction of an organ specific functional MC subtype is needed. hPSC-cSMCs, for instance, could be used as a homeostatic vSMC pool in cases of chronic inflammatory conditions or tissue transplantation (such as human pancreatic islet transplantation), whereas in the case of trauma, highly proliferative hPSC-sSMC would boost the matrix deposition and neovascularization locally *in vivo*. In this context, our simple approach of fabricating 3D vascular organoids of SMC subtypes and ECs and analyzing their phenotype and function is novel and is the first step in designing more complex 3D tissue engineering constructs (by also including organ specific cells and growth factors). Therefore, by fine tuning the phenotypic MC profile we will be able to understand the organotypically differentiated MCs and their functional plasticity and contribution to organ specific health and disease conditions. Accordingly, we propose a flexible, small-scale 3D organoid-like platform consisting of hPSC-SMC/ECs, which seems to be superior to mixed monocells and sole ECs regarding the development of a mature vasculature *in vivo* and is ready-to-use for various tissue engineering applications. Finally, these vascular organoids are a defined *in vitro* model for studying the paracrine interactions between ECs and SMC subtypes that regulate vessel assembly, phenotype modulation, maturation, maintenance and vessel destabilization in a way that mimics the physiological assembly of the normal vasculature and therefore might serve as a platform for drug development, including estimations of compound preclinical toxicity and potential metabolic liability.

## Data Availability Statement

All datasets generated for this study are included in the article/Supplementary Material.

## Ethics Statement

The animal study was reviewed and approved by the Regional Directorate of Rural Economy and Veterinary Medicine, Epirus Region, Greece.

## Author Contributions

MM performed the experiments and drafted the manuscript. DK conceived the study, performed the experiments, and drafted the manuscript. AKy generated the hiPSCs. FB performed the immunohistochemistry. AKa generated the hydrogels. TF conceived the study and interpreted results. CM conceived the study, interpreted results, and edited the manuscript. EB conceived the study, performed the experiments, guided the experiments, interpreted results, and drafted and edited the manuscript.

## Conflict of Interest

The authors declare that the research was conducted in the absence of any commercial or financial relationships that could be construed as a potential conflict of interest.

## Footnotes

1 http://image.bio.methods.free.fr
