# Supplementary material for: Tissue Engineering Using Vascular Organoids From Human Pluripotent Stem Cell Derived Mural Cell Phenotypes
Source: Front Bioeng Biotechnol. 2020 Apr 17;8:278. doi: 10.3389/fbioe.2020.00278 (PMC7182037; doi:10.3389/fbioe.2020.00278)
Supplement: Supplementary file 4 [file Table_1.DOCX]

Supplementary Material

**Legends of the supplementary figures**

**Supplementary Figure 1. Differentiation of hPSCs to contractile and synthetic SMCs A:** hiPSC-cSMCs were cultured in CDM, SDM or CDM supplemented with FGF2 for 48h. Whole-cell lysates were analyzed by SDS-PAGE and immunoblotted with antibodies recognising αSMA, Calponin and SM22α. Quantification of band intensities for each contractile protein is presented in graphs (right). Each bar represents the intensity of the contractile protein normalized to the density of tubulin (loading control) and expressed as fold change relative to cells in CDM. The means±SD were calculated from three independent experiments, ^a^*P*<0.05, ^b^*P*<0.01, ^d^*P*<0.001. **B:** Relative gene expression of *SM22α, CNN1, NG2, PDGFRB, CD105* in hESCs derived -cSMCs, -sSMCs (upper chart) and hiPSCs derived -cSMCs, -sSMCs (lower chart), evaluated by qRT-PCR, from three and two independent experiments respectively. The results are expressed as means± SD, ^b^*P*<0.01. **C:** The medium of hiPSC-cSMCs cultured in CDM, was changed to SDM(0d). Protein expression levels of αSMA, Calponin, SM22α were evaluated on day 2 and 4 by western blot analysis. Tubulin levels serve as loading control. This is a representative image of two independent experiments**. D:** Whole-cell lysates from hESCs, hESC-cSMC, hESC-sSMC and iPSCs, hiPSC-cSMC, hESC-sSMC were analyzed by SDS-PAGE and immunoblotted with antibodies recognizing the pluripotent stem cell markers Nanog and SOX2. This is representative images of two independent experiments.

**Supplementary Figure 2. Maturation of hPSC-cSMCs A:** Chart presenting relative expression of SMMHC in hESC-sSMCs, hESC-cSMCs, and hESC-cSMCs treated with TGFβ1 and heparin for 24h, evaluated by qRT-PCR. The means±SD were calculated from three independent experiments, ^a^P<0.05, NE, not expressed. **B:** Whole-cell lysates from hESC-cSMCs cultured in CDM or in CDM supplemented with TGFβ1 and heparin for 48h, were analyzed by SDS-PAGE and immunoblotted with antibodies recognising αSMA, Calponin and SM22α. Quantification of band intensities for each contractile protein is presented in graph (right). Each bar represents the intensity of the indicated contractile protein normalized to the density of tubulin (loading control) and expressed as fold change relative to cells in CDM. The means±SD were calculated from three independent experiments. **C:** Proliferation assay regarding hESC-cSMCs and hESC-cSMCs, which had been treated with TGFβ1 and heparin for 48h. Medium was changed to DMEM 2%FCS (t=0). Cells were counted after 24h and fold change relative to t=0 is presented in the chart. The means±SD were calculated from three independent experiments. ^a^*P*<0.05.

**Supplementary Figure 3. Functionality of hPSC-SMCs A:** Multipotential capacity. Both hESC-cSMCs and hESC-sSMCs showed osteogenic and chondrogenic differentiation capacities. Osteogenic differentiation resulted in high levels of mineralization (left panels) and alkaline phosphatase production (middle panels) 21d post-induction. Chondrogenic differentiation generated chondropellets rich in sGAGs 15d post-induction (right panels). **B:** Regression analysis. Phase-contrast image of AD-MSCs+ECs derived vascular networks on matrigel at 48h is shown. Image was taken on a Zeiss axiovert 100, microscope and it is representative from two independent experiments. Number of intact meshes representing the vascular network integrity for ECs, AD-MSCs+ECs and hiPSC-MSCs+ECs are shown in the chart. Graph data are expressed as means±SD from two independent experiments. Scale bar, 100μm.

**Supplementary table**

**Supplementary Table1.** Morphometric parameters of the capillary-like structures generated by hPSC-cSMCs+ECs. hPSC sSMCs+ECs and ECs alone on matrigel

| **Morphometric Parameters**  **(n=3 experiments)** | **ECs** | **hESC-cSMCs+ ECs** | **hESC-sSMCs+ ECs** | **ECs** | **hiPSC-cSMCs+ ECs** | **hiPSC-sSMCs+ ECs** |
| --- | --- | --- | --- | --- | --- | --- |
| **Relative number of extremes** | 1.00±0.14 | 1.10±0.08 | 0.94±0.05 | 1.00±0.01 | 0.79±0,02 | 0.84±0,02 |
| **Relative number of nodes** | 1.00±0.05 | 0.89±0.12 | 1.03±0.23 | 1.00±0.05 | 0.83±0,100 | 0.90±0,19 |
| **Relative number of Junctions** | 1.00±0.06 | 0.90±0.11 | 1.00±0.22 | 1.00±0.04 | 0.76±0,09 | 0.90±0,19 |
| **Relative number of master junctions** | 1.00±0.06 | 0.91±0.17 | 0.99±0.18 | 1.00±0.01 | 0.75±0.06 | 0.86±0.14 |
| **Relative number of master segments** | 1.00±0.07 | 0.87±0.14 | 0.99±0.22 | 1.00±0.01 | 0.78±0.07 | 0.87±0.15 |
| **Relative total master segments length** | 1.00±0.08 | 0.90±0.13 | 0.98±0.13 | 1.00±0.02 | 0.77±0.01 | 0.97±0.11 |
| **Relative number of meshes** | 1.00±0.10 | 0.82±0.12 | 1.02±0.33 | 1.00±0.12 | 0.83±0.31 | 1.00±0.34 |
| **Relative total meshes area** | 1.00±0.20 | 0.84±0.24 | 1.01±0.12 | 1.00±0.30 | 1.19±0.38 | 1.65±0.37 |
| **Relative number of pieces** | 1.00±0.04 | 0.93±0.10 | 0.99±0.17 | 1.00±0.03 | 0.72±0.08 | 0.89±0.14 |
| **Relative number of segments** | 1.00±0.06 | 0.87±0.11 | 0.99±0.24 | 1.00±0.04 | 0.76±0.10 | 0.92±0.21 |
| **Relative number of branches** | 1.00±0.05 | 1.07±0.16 | 1.0±0.03 | 1.00±0.02 | 0.85±0.07 | 0.83±0.09 |
| **Relative number of isolated segments** | 1.00±0.65 | 1.30±0.33 | 0.73±0.32 | 1.00±0.18 | 0.75±0.29 | 0.89±0.57 |
| **Relative total length** | 1.00±0.03 | 0.95±0.09 | 0.98±0.09 | 1.00±0.01 | 0.78±0.01 | 0.95±0.06 |
| **Relative total branching length** | 1.00±0.06 | 0.94±0.10 | 0.98±0.11 | 1.00±0.01 | 0.78±0.01 | 0.96±0.09 |
| **Relative total segments length** | 1.00±0.09 | 0.88±0.13 | 0.98±0.15 | 1.00±0.03 | 0.79±0.02 | 0.99±0.12 |
| **Relative total branches length** | 1.00±0.03 | 1.11±0.04 | 0.99±0.01 | 1.00±0.05 | 0.94±0.03 | 0.91±0.04 |
| **Relative total isolated branches length** | 1.00±0.84 | 1.17±0.37 | 0.72±0.50 | 1.00±0.15 | 0.92±0.21 | 0.81±0.65 |
| **Relative branching interval** | 1.00±0.14 | 0.81±0.08 | 0.97±0.14 | 1.00±0.01 | 0.85±0.08 | 1.19±0.01 |
| **Relative mesh index** | 1.00±0.04 | 0.99±0.10 | 0.99±0.06 | 1.00±0.04 | 1.17±0.18 | 1.17±0.08 |
| **Relative mean mesh size** | 1.00±0.26 | 0.97±0.13 | 1.04±0.18 | 1.00±0.19 | 2.09±1.01 | 1.67±0.21 |

**Materials and methods supplementary**

**Table 1.** Conjugated antibodies used in the study

| **Antibody name** | **Clone** | **Manufacturer** |
| --- | --- | --- |
| CD29-PE | MEM-101A | Immunotools, Germany |
| CD73-PE | AD2 | Biolegend, USA |
| CD105-PE | MEM-226 | Immunotools, Germany |
| CD44-PE | MEM-85 | Immunotools, Germany |
| NG2-APC | LHM-2 | R&D systems, USA |
| CD31-PE | MEM-05 | Immunotools, Germany |
| CD34-PE | 4H11[APG] | Immunotools, Germany |
| IgG1-PE | PPV-06 | Immunotools, Germany |
| IgG2-PE | PPV-04 | Immunotools, Germany |
| IgG1-APC | 11711 | R&D systems, USA |

**Table 2.** Primary and secondary antibodies used in the study.

| **Antibody name** | **Clone** | **Manufacturer** |
| --- | --- | --- |
| αSMA | 1A4 | DAKO, USA |
| Calponin | CALP | Dako, Denmark |
| SM22α | Polyclonal | ABCAM, UK |
| Collagen IV | M3F7 | DSHB, USA |
| Fibronectin | P1H11 | DSHB, USA |
| CD31 | JC70A | Dako, Denmark |
| VEGFRII | 55B11 | Cell signaling, USA |
| Von Willebrand Factor | Polyclonal | Dako, Denmark |
| CD34 | QBEnd/10 | ThermoFisher Scientific, USA |
| Nanog | Polyclonal | Cell Signaling, USA |
| Sox2 | 245610 | R&D Systems, USA |
| Tubulin | E7 | DSHB, USA |
| Alexa Fluor® 488 AffiniPure Donkey Anti-Mouse IgG (H+L) | Polyclonal | Jackson ImmunoResearch Laboratories, USA |
| Alexa Fluor® 488 AffiniPure Donkey Anti-Rabbit IgG (H+L) | Polyclonal | Jackson ImmunoResearch Laboratories, USA |
| Alexa Fluor® 594 AffiniPure Donkey Anti-Mouse IgG (H+L) | Polyclonal | Jackson ImmunoResearch Laboratories, USA |
| Rhodamine (TRITC) AffiniPure Donkey Anti-Rabbit IgG (H+L) | Polyclonal | Jackson ImmunoResearch Laboratories, USA |
| Peroxidase AffiniPure Goat Anti-Rabbit IgG (H+L | Polyclonal | Jackson ImmunoResearch Laboratories, USA |
| Peroxidase AffiniPure Goat Anti-Mouse IgG (H+L) | Polyclonal | Jackson ImmunoResearch Laboratories, USA |

**Table 3.** Transcripts and primers used in the study.

| **Transcript name** | **Sequence of Forward (5΄- 3΄) Primer** | **Sequence of Reverse (5΄- 3΄) Primer** |
| --- | --- | --- |
| *NG2* | ACGGAAACGGAAGGTGTCC | CCAGGAAAGGCAACCTTCAAC |
| *SM22a* | CGAAGTGCAGTCCAAAATCGAGAA | AATCACGCCATTCTTCAGCCAGAC |
| *PDGFRβ* | ATCCCCAGTGCCGAGTTAGAAGAC | AGGACAGTGGGCGGTGGGTAGG |
| *SMMHC* | GGCAACGCCAAAACAGTGA | TCAATGTTGGCTCCCACGAT |
| *CD105* | CCCGCACCGATCCAGACCACTCCT | TGTCACCCCTGTCCTCTGCCTCAC |
| CNN | TCATCAAGGCCATCACCAAGT | AGGGTGGACTGCACCTGTGTA |
| *GABDH* | GGTGTGAACCATGAGAAGTATGA | GAGTCCTTCCACGATACCAAAG |
